# Morin Reactivates Nrf2 by Targeting Inhibition of Keap1 to Alleviate Deoxynivalenol-Induced Intestinal Oxidative Damage

**DOI:** 10.3390/ijms26031086

**Published:** 2025-01-27

**Authors:** Gengxiu Zan, Hui He, Xiaofan Wang, Jiayi Zhou, Xiuqi Wang, Huichao Yan

**Affiliations:** State Key Laboratory of Swine and Poultry Breeding Industry, Guangdong Laboratory for Lingnan Modern Agriculture, Guangdong Provincial Key Laboratory of Animal Nutrition Control, National Engineering Research Center for Breeding Swine Industry, College of Animal Science, South China Agricultural University, Guangzhou 510642, China; zangengxiu@163.com (G.Z.); hh15986385009@163.com (H.H.); xxw033@scau.edu.cn (X.W.); 18826487028@163.com (J.Z.); xqwang@scau.edu.cn (X.W.)

**Keywords:** deoxynivalenol, Morin, intestinal stem cells, oxidative stress, Keap1/Nrf2 signaling pathway

## Abstract

As a prevalent mycotoxin found in cereal foods and feed, deoxynivalenol (DON) disrupts the orderly regeneration of intestinal epithelial tissue by interfering with the intracellular antioxidant defense system. However, the potential of mulberry leaf-derived Morin, a natural flavonoid active substance with clearing reactive oxygen species (ROS), to mitigate DON-induced intestinal oxidative damage remains unclear. Our investigation demonstrates that Morin effectively reverses the decline in growth performance and repairs damaged jejunal structures and barrier function under DON exposure. Furthermore, the proliferation and differentiation of intestinal stem cells (ISCs) is enhanced significantly after Morin intervention. Importantly, Morin increases the levels of total antioxidant capacity (T-AOC), superoxide dismutase (SOD), and glutathione peroxidase (GSH-PX) in the serum and jejunal tissue, while reducing the accumulation of ROS and malondialdehyde (MDA). Molecular interaction analysis further confirms that Morin targets inhibition of Keap1 to activate the Nrf2-mediated antioxidant system. In summary, our results suggest that Morin alleviates the oxidative damage induced by DON by regulating the Keap1/Nrf2 pathway, thereby restoring the proliferation and differentiation activity of ISC, which provides new insights into Morin mitigating DON damage.

## 1. Introduction

The intestine functions as the primary site for the absorption and digestion of nutrients, as well as a protective barrier against the intrusion of external toxic substances. Maintaining intestinal epithelial integrity is essential for body health. The intestinal epithelium comprises the villus, facing the lumen and serving as the prominent site for nutrient absorption, and the crypt, an invagination lining along the inner side of the intestine that supplies intestinal stem cells (ISCs) for epithelial replenishment [1]. ISCs are able to differentiate into various types of functional cells [2]. Studies have indicated that ISCs can respond to nutrients or harmful stimuli to alter their own fates, which has a profound influence on intestinal development and regeneration [3].

Deoxynivalenol (DON) is a global mycotoxin with the most widespread and abundant contaminant in feedstuffs and compound feeds, posing a significant risk to food safety and animal production [4]. DON substantially increases intracellular cellular reactive oxygen species (ROS) by impairing mitochondrial function [5,6]. Excessive ROS leads to oxidative stress, causing damage to lipids, DNA, and proteins, ultimately altering cell signaling pathways and triggering apoptosis [7]. Therefore, safeguarding animal intestinal health from the detrimental effects of DON in feed has emerged as a pressing concern within the field of animal husbandry, garnering extensive attention from researchers worldwide.

The dynamic changes of ROS are governed by the nuclear factor E2-related factor 2 (Nrf2) signaling pathway, which is negatively regulated by Kelch-like ECH-associated protein 1 (Keap1) in the cytoplasm under steady-state conditions but translocates to the nucleus to guide the transcription of downstream antioxidant enzymes during oxidative stress [8]. The Keap1/Nrf2 system is recognized as a central cellular defense mechanism against oxidative and xenobiotic stress [9,10]. Sufficient evidence suggests that the Keap1/Nrf2 axis is a key target for resistance to intestinal stress and prevention of inflammatory bowel disease and plays a crucial role in intestinal development and maintenance of normal function [11]. Zhou et al. [12] demonstrated that DON suppresses Nrf2 activity to induce intestinal oxidative damage. On the contrary, several antioxidants (such as l-carnosine) have been shown to activate Nrf2 by interacting with Keap1 to prevent DON-induced intestinal epithelial oxidative damage [13]. Morin, a natural light-yellow pigment extracted from various mulberry plants and many herbal medicines [14], exhibits a wide range of pharmacological properties, including anti-inflammation [15], free radical scavenging [16], and antioxidant effects [17], hence presented as potential methods for the treatment of liver [18] and acute lung injuries [19]. In addition, Morin undergoes efficient metabolism within the body, thereby resolving the problem of conventional antioxidants like BHT residual threats to organism health [20]. Morin consists of the main chain of flavonoids (2-phenyl-1-benzopyran-4-one) with three additional hydroxyl substituents at 2-, 4-, 5- and 7-positions. The antioxidant potential of Morin is primarily attributed to the presence of double bonds and hydroxyl groups (–OH) between C2–C3 atoms, which activate the double bonds at the C3 position and contribute to the electron transport system [14]. Morin has been shown to ameliorate rotenone-induced Parkinson’s disease in mice by improving the antioxidant capacity [21]. Our findings demonstrate that Morin protects intestinal epithelial cells from DON exposure by promoting ISC proliferation and differentiation via modulation of Keap1/Nrf2 signaling in a mouse model, which provides theoretical foundations for utilizing Morin against DON toxicity.

## 2. Results

### 2.1. Morin Reverses the DON-Induced Growth Retardation and Intestinal Barrier Dysfunction in Mice

**Figure 1:**
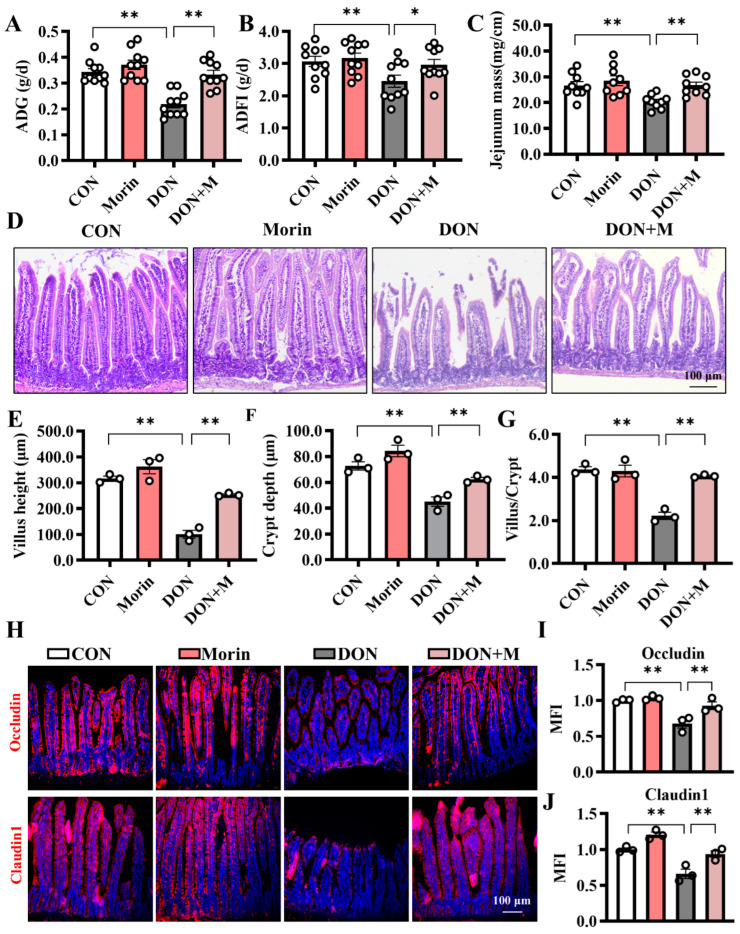
Morin attenuates DON-induced structural damage in mouse jejunum. (**A**) The average daily gain (ADG; *n* = 10); (**B**) the average daily feed intake (ADFI; *n* = 10); (**C**) the jejunum mass (*n* = 9); (**D**) The hematoxylin-eosin (**H**,**E**) staining of the jejunum (200×, *n* = 3); (**E**–**G**) The villus height, crypt depth, and the ratio of villus height to crypt depth (*n* = 3); (**H**–**J**) IHC staining of Occludin, and Claudin1 proteins in the jejunum (200×, *n* = 3). Data were presented as the mean ± SEM. * *p* < 0.05, ** *p* < 0.01.

DON treatment resulted in a significant decrease in ADG and ADFI in mice, while additional Morin reversed this detrimental effect (Figure 1A,B). Additionally, intestinal development was impaired by DON exposure, as indicated by the reduced jejunal mass (Figure 1C). Specifically, the villus height, crypt depth, and villus/crypt ratio were decreased (Figure 1D,G). In addition, DON reduced TEER and protein expressions of Occludin and Claudin1 in the jejunum (Figure 1H–J). Interestingly, supplementation with morin repairs the jejunal structure and barrier function. These results provided evidence that Morin could reverse the growth retardation and protect the integrity of the intestinal epithelium after DON challenge.

### 2.2. Morin Restores the Activity of Jejunal Stem Cells Inhibited by DON Treatment

**Figure 2:**
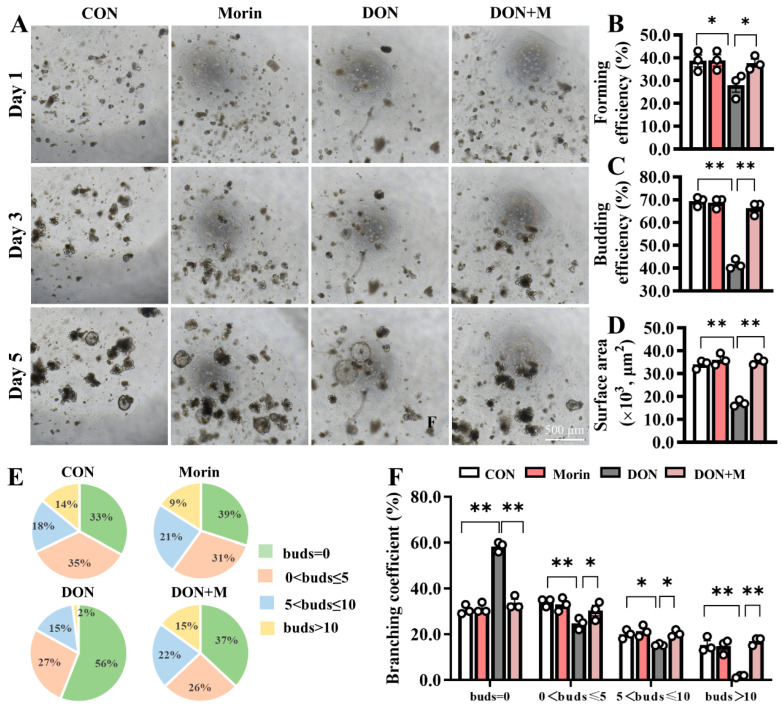
Morin rescues the activation of ISC in DON-exposed mice. (**A**) Images of IOs expanded (40×); Morin increased Ios; (**B**–**D**) The forming efficiency (*n* = 3), budding efficiency (*n* = 3) and surface area (*n* = 15) of IOs; (**E**,**F**) The branching coefficient of IOs (*n* = 3). Data were presented as the mean ± SEM. * *p* < 0.05, ** *p* < 0.01.

Fresh jejunal crypts were isolated from mice in each group and cultured into intestinal organoids (IOs) ex vivo (Figure 2A). In comparison to the CON group, the forming efficiency (Figure 2B), budding efficiency (Figure 2C), surface area (Figure 2D), and branching coefficient (Figure 2E,F) of IOs were significantly reduced in the DON group. However, the IO growth disadvantage was significantly improved in the DON + M group (Figure 2B–F). These results suggest that Morin stimulates ISC expansion.

### 2.3. Morin Improves ISC Proliferation and Suppressed ISC Apoptosis

**Figure 3:**
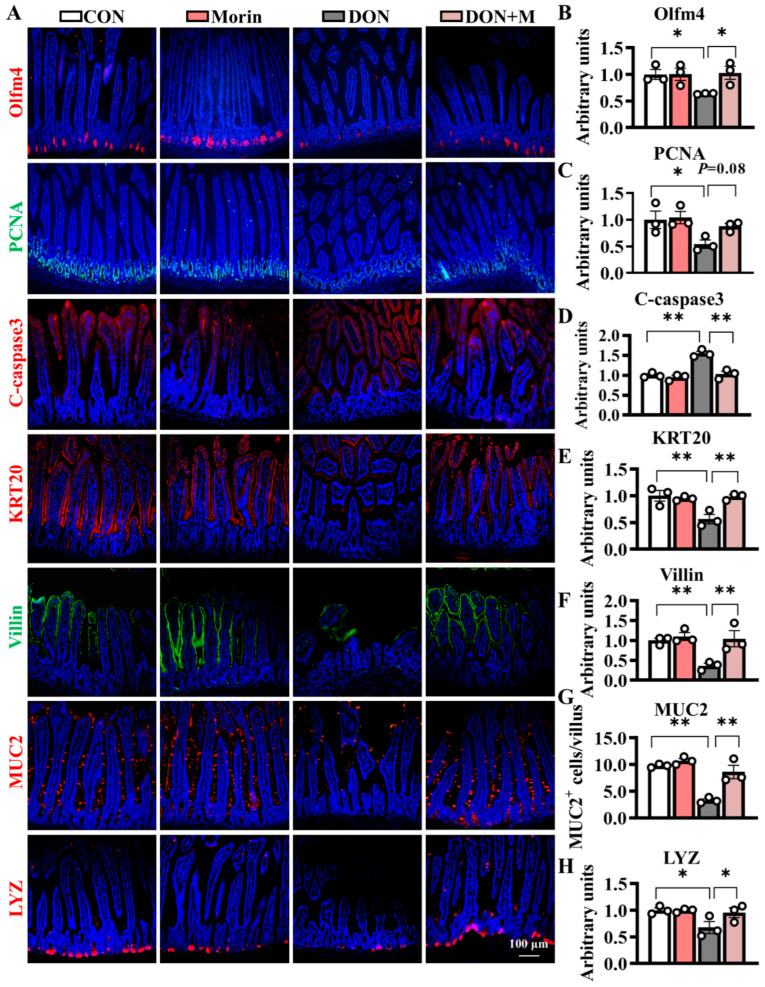
Morin stimulates ISC proliferation and differentiation in mice after DON injury. (**A**–**H**) IHC staining of Olfm4, PCNA, C-caspase3, KRT20, Villin, MUC2 and LYZ proteins in the jejunum (200×, *n* = 3). Data were presented as the mean ± SEM. * *p* < 0.05, ** *p* < 0.01.

Compared with the control group, DON-treated mice exhibited significant suppression of proliferative cell marker PCNA, as well as increased expression levels of apoptosis cell marker C-caspase3. Correspondingly, the immunofluorescence signal intensities of the Olfm4 (an ISC marker) and PCNA significantly downregulated in the DON group, while those of the C-caspase3 significantly upregulated. The Morin supplementation notably mitigated DON-induced ISC dysfunction and suppressed cell apoptosis (Figure 3A–D). Hence, Morin could improve ISC proliferation and apoptosis under DON exposure.

### 2.4. Morin Promotes ISC Differentiation Under DON Exposure

DON significantly hindered ISC differentiation, as evidenced by reduced expression of differentiation cell marker KRT20 and absorptive cell marker Villin. The populations of MUC2^+^ (goblet cell marker) and LYZ^+^ (Paneth cell marker) cells also decreased following DON treatment. Importantly, Morin rescued the ISC differentiation as demonstrated by significantly higher fluorescence signal intensities of KRT20 and Villin, as well as increased numbers of MUC2^+^ and LYZ^+^ cells. These findings suggest that Morin has the potential to promote ISC differentiation in response to DON exposure (Figure 3A,E–H).

### 2.5. Morin Enhances Antioxidant Capacity in DON-Treated Mice

**Figure 4:**
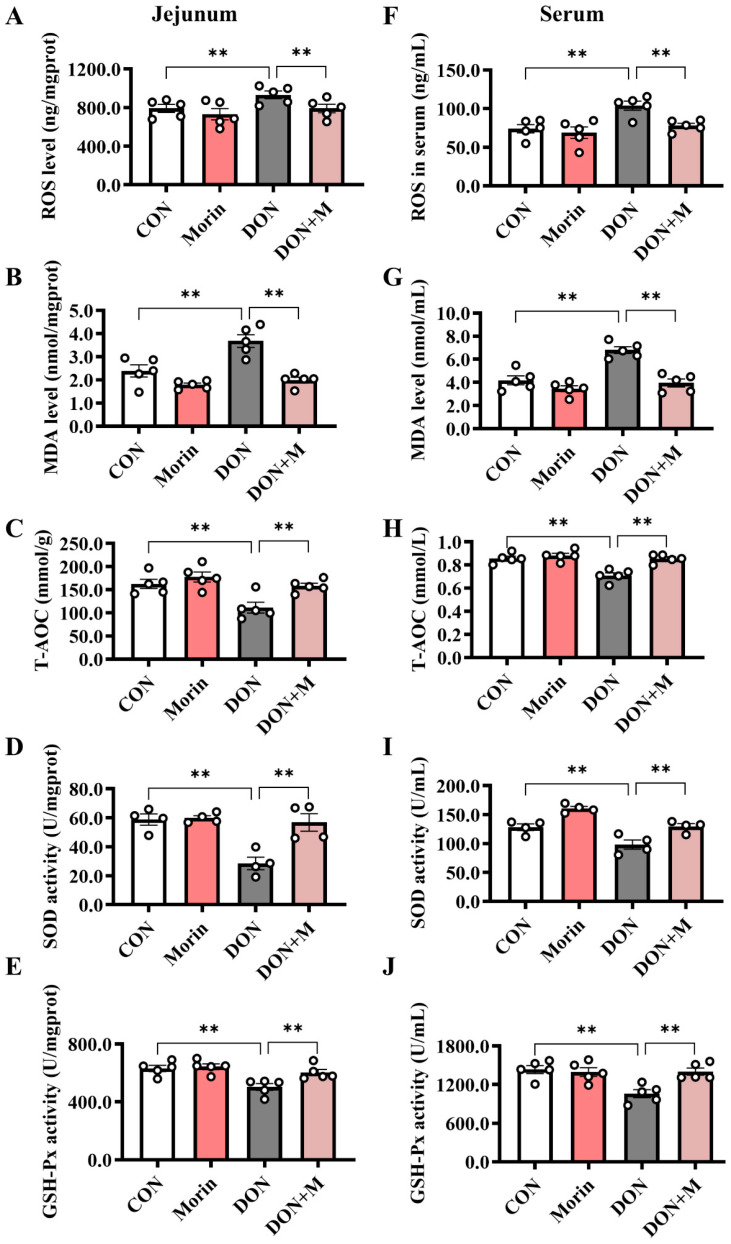
Morin enhances antioxidant capacity in DON-treated mice. (**A**–**E**) The level of ROS (*n* = 5), MDA (*n* = 5), T-AOC (*n* = 5), SOD (*n* = 4), and GSH-PX (*n* = 5) in the jejunum. (**F**–**J**) The level of ROS (*n* = 5), MDA (*n* = 5), T-AOC (*n* = 5), SOD (*n* = 4), and GSH-PX (*n* = 5) in the serum. Data were presented as the mean ± SEM. ** *p* < 0.01.

Compared with the control group, DON significantly increased the levels of ROS and MDA and decreased the levels of T-AOC, SOD, and GSH-Px in both the jejunum and serum. While Morin enhanced antioxidant capacity after DON injury (Figure 4A–J).

### 2.6. Morin Reactivates the Nrf2 Signaling Pathway upon DON Treatment

**Figure 5:**
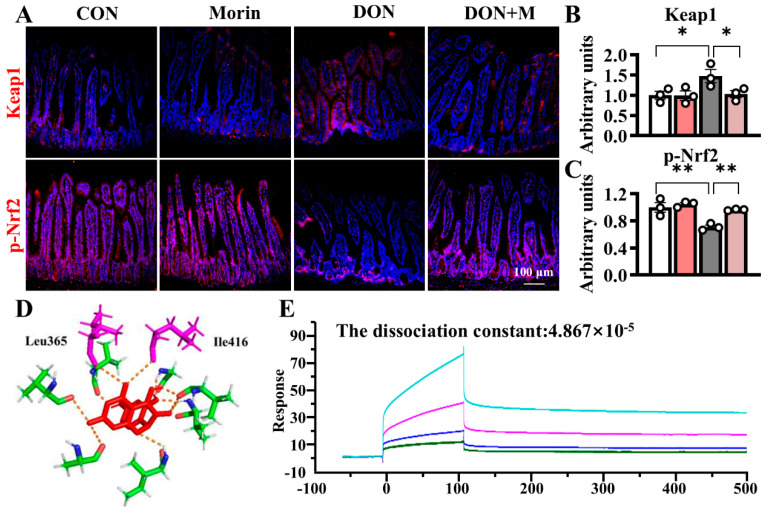
Morin promotes the jejunal Keap1/Nrf2 signaling in mice after DON exposure. (**A**–**C**) IHC staining of Keap1 and p-Nrf2 proteins in the jejunum (200×) (*n* = 3); (**D**,**E**) Morin docking with Keap1 molecule. Data were presented as the mean ± SEM. * *p* < 0.05, ** *p* < 0.01.

DON increased Keap1 expression in the jejunum and suppressed p-Nrf2 expression in the jejunum. Morin inhibited Keap1 expression and reactivated Nrf2 signaling under DON exposure (Figure 5A–C). Molecular docking analysis suggested that Morin occupied the binding sites between Nrf2 and Keap1, overlapping with ETGE and DLG amino acid motifs (Ile416 and Leu365), with a binding energy of −6.94 kcal/mol. The Biacore confirmed that Morin could bind to Keap1, with a dissociation constant of 4.867 × 10^−5^ (Figure 5D–E), indicating strong binding affinity between Morin and Keap1.

## 3. Discussion

DON is widely presented in nature and easily contaminates cereals and animal feed, which causes substantial economic loss [22]. In addition, DON accumulation can seriously damage the intestinal epithelial structure, induce diarrhea, and increase the risk of pathogenic infections [23]. Previous findings indicated that exposure to DON leads to an imbalanced gut redox due to increased ROS production and weakened antioxidant defense systems [24]. Therefore, improving intestinal antioxidant capacity seems to be a potential strategy to relieve DON damage. Morin, as a natural compound with high antioxidant properties, has been proven to effectively protect the intestinal tract of mice from STb damage [25]. In the current study, we evaluated the efficacy of Morin in protecting the intestinal epithelium from DON-induced oxidative stress damage, aiming to develop a novel intestinal protective agent.

Some studies have shown that ingestion of feed contaminated with DON can lead to damage in the intestinal tract and decrease animal growth performance. Previous research has shown that dietary exposure to DON reduces growth performance while causing intestinal damage [26,27]. Consistent with these findings, our results confirmed that administration of DON in mice caused growth retardation and intestinal barrier dysfunction, which was reversed by Morin through increased ADG, ADFI, villus height, and intestinal integrity. Considering the protective effect of Morin on barrier function, we assessed the replenishment potentials of the ISC expansion. The data indicate that Morin was able to reactivate ISCs and accelerate the repair process of the intestinal epithelium, which were supported by the increased expressions of proliferation marker (Olfm4 and PCNA) and reduced apoptosis marker (C-caspase3) in crypts. Moreover, Morin facilitated ISC differentiation into functional lineages such as Paneth cells, goblet cells, etc., to strengthen intestinal functions following DON insults. Notably, goblet cells (MUC2) and Paneth cells (LYZ) could protect the integrity of intestinal epithelium from endogenous toxins-caused damage [25]. After the Morin supplement, the reconstruction of the ISC-absorptive cell axis contributes to the rapid intake of nutrients, while the ISC–secretory cell axis effectively protects the epithelium.

Oxidative stress is thought to be an important mechanism by which DON induces toxic effects such as hepatotoxicity, immunotoxicity, and enterotoxicity. DON exposure triggers ROS production, increases MDA levels, inhibits the expression of antioxidant enzymes (SOD, T-AOC, GSH-Px), and ultimately leads to oxidative stress and intestinal barrier dysfunction [28,29]. Morin has been identified as an effective antioxidant capable of mitigating ROS accumulation and safeguarding cells from oxidative stress [30]. Our results demonstrate that Morin significantly reduced the accumulation of ROS and MDA in serum and jejunal tissues while enhancing the activity of antioxidant enzymes (SOD, T-AOC, GSH-Px) under DON exposure. This uncovered mechanism aligns with previous reports indicating that Morin alleviates oxidative stress induced by midbrain carotid artery occlusion in rats by reducing MDA expression and increasing the activity of antioxidant enzymes SOD and GSH-Px [31].

DON causes intestinal redox imbalance characterized by increased production of ROS and weakened antioxidant defenses. The Keap1/Nrf2 signaling pathway is the essential defense mechanism against oxidative stress [32]. Under normal conditions, Nrf2 is captured by Keap1 in the cytoplasm and continuously degraded by ubiquitin [33,34]. However, when confronted with oxidative stress, the accumulation of ROS disrupts intracellular redox balance, leading to inhibition of Nrf2 expression and its downstream antioxidant enzymes [21]. Our current findings indicated that DON upregulated the expression of Keap1 and downregulated the expression of p-Nrf2. Consequently, supplementation with Morin inhibits Keap1 activity and reactivates the p-Nrf2 signaling pathway. This discovery confirmed previous findings that certain nutritional compounds can mitigate DON-induced oxidative damage in ISC through the Keap1/Nrf2 signaling pathway [35]. Similarly, Morin has been shown to provide cellular protection in C2C12 myoblasts against H_2_O_2_-induced DNA damage and cytotoxicity by suppressing intracellular ROS production and activating the Nrf2/HO-1 pathway [30]. Furthermore, molecular docking analysis indicated that Morin possesses a strong binding site for Keap1, providing new molecular evidence for the interaction between Morin and Keap1. Our data highlight that Morin alleviates DON-induced oxidative damage in the small intestine by targeting Keap1 for degradation and activating Nrf2.

**Figure 6:**
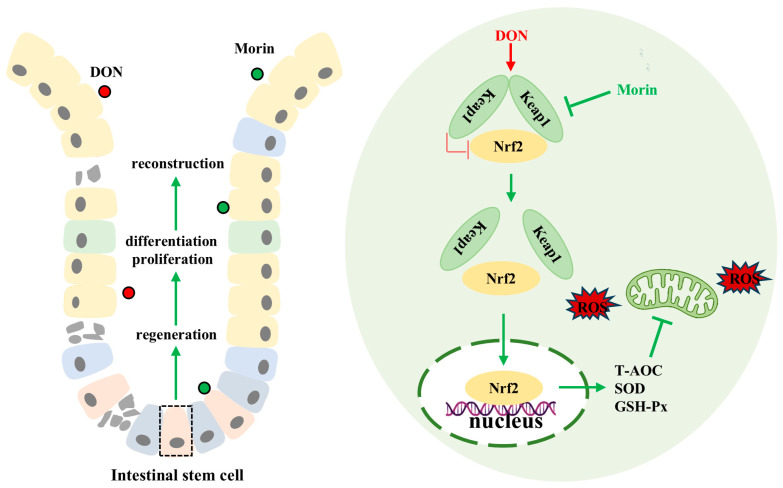
Schematic representation of the mechanisms of Morin protecting against DON. Morin reactivates Nrf2 by targeting the inhibition of Keap1 to alleviate deoxynivalenol-induced intestinal oxidative damage.

In conclusion, our findings demonstrate that Morin enhances antioxidant capacity, promotes ISC proliferation and inhibits apoptosis, preserves intestinal structural and functional integrity, and mitigates DON-induced intestinal injury through modulation of the Keap1/Nrf2 signaling pathway (Figure 6). The function of food health agents requires the joint verification of multiple models, so the co-evaluation of the biological activity of Morin using different varieties of animal or human cells is the focus of our future research.

## 4. Materials and Methods

### 4.1. Laboratory Equipment

The Ussing Chamber (C4300/VCC MC6 Plus, Beijing KingTech Technology Co., Ltd., Beijing, China) was used to detect intestinal trans-epithelial electrical resistance (TEER) values; the microscope (Ti2-U, Nikon, Tokyo, Japan) was used to capture representative images of immunofluorescence, Hematoxylin-Eosin (H&E) Staining, and organoid growth; the microplate reader (Multiskan SkyHigh, Thermo Fisher, Waltham, MA, USA) was used to measure absorbance; the carbon dioxide incubator (SCO6WE-2, Guangzhou south biomedical, Guangzhou, China) was used to cultivate organoids and the Biacore (Biacore 8K, GE, Boston, MA, USA) was used to verify the binding reaction of Morin and Keap1.

### 4.2. Reagents

Antibodies against Claudin1 (#bs-10008R, Bioss, Beijing, China), Cleaved caspase-3 (C-Caspase3, # 341034, Zen Bioscience, Chengdu, China), Keap1 (#R26935, Zen Bioscience, Chengdu, China), Keratin 20 (KRT20, #13063, Cell Signaling Technology, Boston, MA, USA), LYZ (#A0099, DAKO, Copenhagen, Denmark), Mucin2 (MUC2, #A14659, Abclonal, Wuhan, China), Occludin (#502601, Zen BioScience, Chengdu, China), Olfm4 (#39141, Cell Signaling Technology, Boston, MA, USA), PCNA (#200947-2E1, Zen Bioscience, Chengdu, China), p-Nrf2 (#381559, Zen Bioscience, Chengdu, China), Villin (#sc-58897, Santa Cruz, TX, USA), and β-actin (#600149, Zen BioScience, Chengdu, China), as well as anti-rabbit IgG (#511203, Zen BioScience, Chengdu, China) and anti-mouse IgG (#511103, Zen BioScience, Chengdu, China), were used for Western blotting or immunohistochemistry.

### 4.3. Animals and Treatments

A total of 40 healthy 4-week-old C57BL/6 male mice with similar body weight (BW) were divided into four groups (*n* = 10): Control (PBS, physiological saline), Morin (#M4008, Sigma, St. Louis, MO, USA) (10 mg/kg BW Morin), DON (#047M4047, Sigma) (3 mg/kg BW DON), and DON + Morin (DON + M; 10 mg/kg BW Morin + 3 mg/kg BW DON) groups. The mice were housed under 25 °C, 60% relative humidity, and a regular 12 h:12 h light:dark cycle and continuously gavage-fed with each treatment for 10 days and then euthanized on the 11th day using CO_2_ inhalation. Each group of mice was kept free to feed and drink, and their body weight, food intake, and water intake were recorded to assess the average daily gain (ADG), average daily feed intake (ADFI), and average daily water intake (ADWI). All the experiments were approved by the Animal Ethics Committee of South China Agricultural University (2023F327, Guangzhou, China).

### 4.4. Crypt Isolation and Culture

As described in the previous study [36], 5 cm jejunum mice were longitudinally excised and washed with pre-cooled Dulbecco phosphate-buffered saline (DPBS). The intestinal tissues were then placed in Petri dishes, and the villi were removed with glass slides. After PBS rinsing, the tissue was washed with DPBS, cut into 5 × 5 mm segments, and transferred to a 50 mL centrifuge tube (Jet BioFit, Guangzhou, China) with 20 mL soaking buffer containing 30 mM disodium ethylenediaminetetraacetate and 1.5 mM dithiothreitol (DTT; Sigma-Aldrich, St. Louis, MO, USA). Debris and villi were decanted and discarded while crypts were collected and resuspended in Matrigel and grown in the medium at 37 °C. The ISC medium containing N2 supplement (Invitrogen, Carlsbad, CA, USA), B27 supplement (Invitrogen), N-acetylcysteine (Sigma-Aldrich), recombinant murine epidermal growth factor (EGF; PeproTech, Rocky Hill, NJ, USA), recombinant murine noggin (PeproTech, Cranbury, NJ, USA), recombinant human Rspo1 (R&D Systems, Minneapolis, MN, USA), nicotinamide (Sigma-Aldrich, St. Louis, MO, USA), a p160ROCK inhibitor (Y27632; Stemgent, Cambridge, MA, USA), a transforming growth factor β (TGFβ) receptor inhibitor (LY2157299; Selleck, Houston, TX, USA), a p38 mitogen-activated protein (MAP) kinase inhibitor (SB202190; Sigma-Aldrich, St. Louis, MO, USA), and a glycogen synthase kinase 3 inhibitor (GSK3i; CHIR99021; Stemgent, Cambridge, MA, USA).

### 4.5. H&E Staining

Jejunal tissues were fixed in 4% paraformaldehyde and then washed with PBS. Subsequently, the tissues underwent dehydration with alcohol and hardening with xylene before being embedded in paraffin. The embedded tissues were then sliced and subjected to H&E staining. Villus height and crypt depth were measured using ImageJ (v2.3.0) software (National Institute of Health, Bethesda, MD, USA).

### 4.6. Immunohistochemistry

The jejunal sections were incubated with primary antibodies overnight at 4 °C and the Cy3- or FITC-conjugated secondary antibody (Jackson Laboratory, Jackson, MS, USA) at room temperature for 2 h. The nuclei were stained with 4′,6-diamidino-2-phenylindole (DAPI, Sigma-Aldrich) for 10 min. Images were captured with a microscope, and ImageJ (v2.3.0) software (National Institute of Health, Bethesda, MD, USA) was used to analyze positive cell numbers and fluorescence signal intensity.

### 4.7. Measurement of Oxidative–Antioxidant Parameters

The sample (serum or tissue homogenate) and the standard were injected into the test plate, and the corresponding reagents were added in sequence according to the instructions provided by the kit. Then, the absorbance was measured at 532 nm with a microplate reader. The concentration and absorbance of the standard were used to draw the regression curve, and finally, the sample absorbance was brought into the formula to calculate the ROS, total antioxidant capacity (T-AOC), superoxide dismutase (SOD), glutathione peroxidase (GSH-PX), and malondialdehyde (MDA) concentration.

### 4.8. Molecular Docking Analysis

The molecular structures of Morin (ZINC2040854) and Keap1 (4IFJ) were downloaded from the ZINC database (http://zinc.docking.org/, accessed on 6 March 2024) and Protein Data Bank (https://www.rcsb.org/, accessed on 6 March 2024), respectively, and loaded into AutoDock for analysis. PyMOL was used to draw three-dimensional images of Morin and Keap1 combined. Finally, the Keap1 protein (11981-HNCB, Sino Biological, Beijing, China) is attached to the chip, and its binding to Morin is tested by the Biacore (Biacore 8K, GE, Boston, MA, USA) instrument.

### 4.9. Statistical Analysis

GraphPad Prism 8.0 software was used for statistical analysis. All data are presented as the mean ± SEM. Group comparisons were conducted using one-way ANOVA and Dunnett’s post-test, and significance levels were denoted by * *p* < 0.05, ** *p* < 0.01, while statistical tendencies were indicated by 0.05 ≤ *p*-values < 0.10.

## Data Availability

Data will be made available on request.

## References

[1] Clevers H., Loh K.M., Nusse R. (2014). An integral program for tissue renewal and regeneration: Wnt signaling and stem cell control. Science.

[2] Yan K.S., Janda C.Y., Chang J., Zheng G.X.Y., Larkin K.A., Luca V.C., Chia L.A., Mah A.T., Han A., Terry J.M. (2017). Non-equivalence of Wnt and R-spondin ligands during Lgr5+ intestinal stem-cell self-renewal. Nature.

[3] Zhou J.-Y., Lin H.-L., Wang Z., Zhang S.-W., Huang D.-G., Gao C.-Q., Yan H.-C., Wang X.-Q. (2020). Zinc L-Aspartate enhances intestinal stem cell activity to protect the integrity of the intestinal mucosa against deoxynivalenol through activation of the Wnt/β-catenin signaling pathway. Environ. Pollut..

[4] Kang R., Li R., Dai P., Li Z., Li Y., Li C. (2019). Deoxynivalenol induced apoptosis and inflammation of IPEC-J2 cells by promoting ROS production. Environ. Pollut..

[5] Hou S., Ma J., Cheng Y., Wang H., Sun J., Yan Y. (2023). The toxicity mechanisms of DON to humans and animals and potential biological treatment strategies. Crit. Rev. Food Sci. Nutr..

[6] Wang S., Wu K., Xue D., Zhang C., Rajput S.A., Qi D. (2021). Mechanism of deoxynivalenol mediated gastrointestinal toxicity: Insights from mitochondrial dysfunction. Food Chem. Toxicol..

[7] Chen X., Zhu N., Wu Y., Zhang Y., Zhang Y., Jin K., Zhou Z., Chen G., Wang J. (2024). Withaferin A, a natural thioredoxin reductase 1 (TrxR1) inhibitor, synergistically enhances the antitumor efficacy of sorafenib through ROS-mediated ER stress and DNA damage in hepatocellular carcinoma cells. Phytomedicine.

[8] Kang M.-I., Kobayashi A., Wakabayashi N., Kim S.-G., Yamamoto M. (2004). Scaffolding of Keap1 to the actin cytoskeleton controls the function of Nrf2 as key regulator of cytoprotective phase 2 genes. Proc. Natl. Acad. Sci. USA.

[9] Au W.H., Miller-Fleming L., Sanchez-Martinez A., Lee J.A., Twyning M.J., A Prag H., Raik L., Allen S.P., Shaw P.J., Ferraiuolo L. (2024). Activation of the Keap1/Nrf2 pathway suppresses mitochondrial dysfunction, oxidative stress, and motor phenotypes in *C9orf72* ALS/FTD models. Life Sci. Alliance.

[10] Kensler T.W., Wakabayashi N., Biswal S. (2007). Cell Survival Responses to Environmental Stresses Via the Keap1-Nrf2-ARE Pathway. Annu. Rev. Pharmacol. Toxicol..

[11] Piotrowska M., Swierczynski M., Fichna J., Piechota-Polanczyk A. (2021). The Nrf2 in the pathophysiology of the intestine: Molecular mechanisms and therapeutic implications for inflammatory bowel diseases. Pharmacol. Res..

[12] Zhou J., Lin H., Qin Y., Li X., Gao C., Yan H., Wang X. (2021). l-Carnosine Protects Against Deoxynivalenol-Induced Oxidative Stress in Intestinal Stem Cells by Regulating the Keap1/Nrf2 Signaling Pathway. Mol. Nutr. Food Res..

[13] Rajput S.A., Liang S.-J., Wang X.-Q., Yan H.-C. (2021). Lycopene Protects Intestinal Epithelium from Deoxynivalenol-Induced Oxidative Damage via Regulating Keap1/Nrf2 Signaling. Antioxidants.

[14] Caselli A., Cirri P., Santi A., Paoli P. (2016). Morin: A Promising Natural Drug. Curr. Med. Chem..

[15] Yu S., Liu X., Yu D., Changyong E., Yang J. (2020). Morin Protects LPS-Induced Mastitis via Inhibiting NLRP3 Inflammasome and NF-κB Signaling Pathways. Inflammation.

[16] Lee M.H., Cha H.-J., Choi E.O., Han M.H., Kim S.O., Kim G.-Y., Hong S.H., Park C., Moon S.-K., Jeong S.-J. (2017). Antioxidant and cytoprotective effects of morin against hydrogen peroxide-induced oxidative stress are associated with the induction of Nrf-2-mediated HO-1 expression in V79-4 Chinese hamster lung fibroblasts. Int. J. Mol. Med..

[17] Rizvi F., Mathur A., Krishna S., Siddiqi M.I., Kakkar P. (2015). Suppression in PHLPP2 induction by morin promotes Nrf2-regulated cellular defenses against oxidative injury to primary rat hepatocytes. Redox Biol..

[18] Li X., Yao Q., Huang J., Jin Q., Xu B., Chen F., Tu C. (2019). Morin Hydrate Inhibits TREM-1/TLR4-Mediated Inflammatory Response in Macrophages and Protects Against Carbon Tetrachloride-Induced Acute Liver Injury in Mice. Front. Pharmacol..

[19] Cai B., Gan X., He J., He W., Qiao Z., Ma B., Han Y. (2018). Morin attenuates cigarette smoke-induced lung inflammation through inhibition of PI3K/AKT/NF-κB signaling pathway. Int. Immunopharmacol..

[20] Amorati R., Foti M.C., Valgimigli L. (2013). Antioxidant Activity of Essential Oils. J. Agric. Food Chem..

[21] Ishola I., Awogbindin I., Olubodun-Obadun T., Oluwafemi O., Onuelu J., Adeyemi O. (2022). Morin ameliorates rotenone-induced Parkinson disease in mice through antioxidation and anti-neuroinflammation: Gut-brain axis involvement. Brain Res..

[22] Li X., Gou F., Zhu J., Lin Q., Yu M., Tu X., Hong Q., Hu C. (2024). Deoxynivalenol induced intestinal barrier injury, mitochondrial dysfunction and calcium overload by inositol 1,4,5-triphosphate receptors (IP3Rs)-mitochondrial calcium uniporter (MCU) calcium axis. Sci. Total Environ..

[23] Zhou B., Xiao K., Guo J., Xu Q., Xu Q., Lv Q., Zhu H., Zhao J., Liu Y. (2024). Necroptosis contributes to the intestinal toxicity of deoxynivalenol and is mediated by methyltransferase SETDB1. J. Hazard. Mater..

[24] Mishra S., Kapoor R., Sushma, Kanchan S., Jha G., Sharma D., Tomar B., Rath S.K. (2024). Deoxynivalenol Induces Drp-1-Mediated Mitochondrial Dysfunction via Elevating Oxidative Stress. Chem. Res. Toxicol..

[25] Zhou J.-Y., Xie W.-W., Hu T.-C., Wang X.-F., Yan H.-C., Wang X.-Q. (2024). Mulberry Leaf-Derived Morin Activates β-Catenin by Binding to Frizzled7 to Promote Intestinal Stem Cell Expansion upon Heat-Stable Enterotoxin b Injury. J. Agric. Food Chem..

[26] Alharbi K., Ekesi N., Hasan A., Asnayanti A., Liu J., Murugesan R., Ramirez S., Rochell S., Kidd M.T., Alrubaye A. (2024). Deoxynivalenol and Fumonisin Predispose Broilers to Bacterial Chondronecrosis with Osteomyelitis Lameness. Poult. Sci..

[27] Miao C., Wu Z., Sun Y., Cao Z. (2024). Deoxynivalenol Induces Intestinal Epithelial Barrier Damage through RhoA/ROCK Pathway-Mediated Apoptosis and F-Actin-Associated Tight Junction Disruption. J. Agric. Food Chem..

[28] Chenna S., Chenna S., Koopman W.J.H., Koopman W.J.H., Prehn J.H.M., Prehn J.H.M., Connolly N.M.C., Connolly N.M.C. (2022). Mechanisms and mathematical modeling of ROS production by the mitochondrial electron transport chain. Am. J. Physiol. Physiol..

[29] Yang Y., Tian Z., Ding Y., Li X., Zhang Z., Yang L., Zhao F., Ren F., Guo R. (2018). EGFR-Targeted Immunotoxin Exerts Antitumor Effects on Esophageal Cancers by Increasing ROS Accumulation and Inducing Apoptosis via Inhibition of the Nrf2-Keap1 Pathway. J. Immunol. Res..

[30] Lee M.H., Han M.H., Lee D.-S., Park C., Hong S.-H., Kim G.-Y., Hong S.H., Song K.S., Choi I.-W., Cha H.-J. (2017). Morin exerts cytoprotective effects against oxidative stress in C2C12 myoblasts via the upregulation of Nrf2-dependent HO-1 expression and the activation of the ERK pathway. Int. J. Mol. Med..

[31] Chen Y., Li Y., Xu H., Li G., Ma Y., Pang Y.J. (2017). Morin mitigates oxidative stress, apoptosis and inflammation in cerebral ischemic rats. Afr. J. Tradit. Complement. Altern. Med..

[32] Magesh S., Chen Y., Hu L. (2012). Small Molecule Modulators of Keap1-Nrf2-ARE Pathway as Potential Preventive and Therapeutic Agents. Med. Res. Rev..

[33] Bellezza I., Giambanco I., Minelli A., Donato R. (2018). Nrf2-Keap1 signaling in oxidative and reductive stress. Biochim. Biophys. Acta (BBA) Mol. Cell Res..

[34] Loboda A., Damulewicz M., Pyza E., Jozkowicz A., Dulak J. (2016). Role of Nrf_2_/HO_-1_ system in development, oxidative stress response and diseases: An evolutionarily conserved mechanism. Cell. Mol. Life Sci..

[35] Zhu C., Liang S., Zan G., Wang X., Gao C., Yan H., Wang X., Zhou J. (2023). Selenomethionine Alleviates DON-Induced Oxidative Stress via Modulating Keap1/Nrf2 Signaling in the Small Intestinal Epithelium. J. Agric. Food Chem..

[36] Zhou J.-Y., Zhang S.-W., Lin H.-L., Gao C.-Q., Yan H.-C., Wang X.-Q. (2019). Hydrolyzed wheat gluten alleviates deoxynivalenol-induced intestinal injury by promoting intestinal stem cell proliferation and differentiation via upregulation of Wnt/β-catenin signaling in mice. Food Chem. Toxicol..

